# Monocarboxylate Transporters 1–4 in NSCLC: MCT1 Is an Independent Prognostic Marker for Survival

**DOI:** 10.1371/journal.pone.0105038

**Published:** 2014-09-16

**Authors:** Marte Eilertsen, Sigve Andersen, Samer Al-Saad, Yury Kiselev, Tom Donnem, Helge Stenvold, Ingvild Pettersen, Khalid Al-Shibli, Elin Richardsen, Lill-Tove Busund, Roy M. Bremnes

**Affiliations:** 1 Department of Clinical Medicine, University of Tromso, Tromso, Norway; 2 Department of Oncology, University Hospital of North Norway, Tromso, Norway; 3 Department of Medical Biology, University of Tromso, Tromso, Norway; 4 Department of Clinical Pathology, University Hospital of North Norway, Tromso, Norway; 5 Department of Pathology, Nordland Central Hospital, Bodo, Norway; 6 Department of Pharmacy, University of Tromso, Tromso, Norway; University Health Network, Canada

## Abstract

**Introduction:**

Monocarboxylate transporters (MCTs) 1–4 are lactate transporters crucial for cancers cells adaption to upregulated glycolysis. Herein, we aimed to explore their prognostic impact on disease-specific survival (DSS) in both cancer and tumor stromal cells in NSCLC.

**Methods:**

Tissue micro arrays (TMAs) were constructed, representing both cancer and stromal tumor tissue from 335 unselected patients diagnosed with stage I–IIIA NSCLC. Immunohistochemistry was used to evaluate the expression of MCT1-4.

**Results:**

In univariate analyses; ↓MCT1 (P = 0.021) and ↑MCT4 (P = 0.027) expression in cancer cells, and ↑MCT1 (P = 0.003), ↓MCT2 (P = 0.006), ↓MCT3 (P = 0.020) expression in stromal cells correlated significantly with a poor DSS. In multivariate analyses; ↓MCT1 expression in cancer cells (HR: 1.9, CI 95%: 1.3–2.8, P = 0.001), ↓MCT2 (HR: 2.4, CI 95%: 1.5–3.9, P<0.001), ↓MCT3 (HR: 1.9, CI 95%: 1.1–3.5, P = 0.031) and ↑MCT1 expression in stromal cells (HR: 1.7, CI 95%: 1.1–2.7, P = 0.016) were significant independent poor prognostic markers for DSS.

**Conclusions:**

We provide novel information of MCT1 as a candidate marker for prognostic stratification in NSCLC. Interestingly, MCT1 shows diverging, independent prognostic impact in the cancer cell and stromal cell compartments.

## Introduction

Non-small cell lung cancer (NSCLC) is a major cause of cancer deaths in the Western World, with a 5-year survival still as low as 16% in the United States [1]. The latter is due to late symptoms and lack of early detection measures. New and better predictive and prognostic markers in NSCLC are highly warranted.

Hypoxia is a common feature of solid tumors [2], and our research group has previously published articles on hypoxic markers in NSCLC [3]–[6]. A necessary metabolic adaption to hypoxia is a switch to energy generation by glycolysis. In addition, malignant cells in general even seem to prefer glycolysis despite the presence of oxygen (“Warburg effect”) [7]. The cancer cells' ability to switch to glycolysis is believed to represent a growth advantage, since the oxygen availability in a tumor can fluctuate over time [8]. However, glycolysis also increases lactic acid production. To avoid intracellular acidification and apoptosis, glycolytic cells must sustain lactate homeostasis. Several transporters are involved in this process including monocarboxylate transporters (MCT) 1–4 [9]. MCT1-4 are trans-membrane symporters involved in lactate and pyruvate transportation. MCT1 and MCT4 are located in the cell membrane. MCT4 exports lactate, while MCT1 can facilitate both import and export depending on the pH gradient [10]. The potential roles of MCT2 and MCT3 in cancers are less studied. MCT2 is reported to be expressed in the mitochondrial membrane, where it is involved in the import of pyruvate following lactate oxidation [11]. MCT3 export lactate, but is only reported to be expressed in retinal pigment epithelium and choroid plexus epithelium [9]. Lactate homeostasis can also be sustained through metabolic co-operation between cancer cells and tumor stroma cells [11], [12]. This theory of metabolic co-operation is based on the observation that cancer cells express proteins involved in anaerobic glycolysis (like GLUT1), while stromal cells express complementary proteins involved in lactate oxidation.

Although energy metabolism has been a rather unexploited field in cancer treatment, effectors of energy metabolism are intriguing targets of therapy [13]. The expression of MCTs and their functional role in normal tissue is well characterized, but the transporter expression and role in different cancers has just recently started to be investigated [9]. Due to the recent observation that MCTs may play a central part in tumor biology, and that MCT1 is considered as a potential target in cancer treatment, we aimed to explore the prognostic impact of MCT1–4 on disease specific survival (DSS) in both cancer and tumor stromal cells from NSCLC patients. In addition, we investigated the potential synergetic impact of co-expression of metabolic markers in NSCLC.

## Materials and Methods

### Ethics statement

The Norwegian Data Inspectorate and The Regional Committee for Medical and Health Research Ethics Nord (Nordland, Troms and Finnmark) have approved the study. Patient records/information was anonymized and de-identified prior to analysis. The need for consent was specifically waived by The Regional Committee for Research Ethics, since the study was retrospective with more than half of the patients deceased.

### Patients

Primary tumor tissue samples from 371 patients diagnosed with NSCLC stage I-IIIA at the University Hospital of Northern Norway and Nordland Central Hospital from 1990 to 2004 were collected retrospectively. Thirty-six patients were excluded from the cohort. Exclusion from the study was due to radiotherapy or chemotherapy prior to surgery (n = 10), other malignancy within the 5 years prior to diagnosis (n = 13) or inadequate paraffin-embedded fixed tissue blocks (n = 13). A representative cohort of 335 patients was included in the study, and complete demographic and clinicopathological data were obtained retrospectively. An anonymised database was established. Staging of the tumors was done according to the World Health Organization Guidelines [14]. Two experienced pathologists reviewed all primary tissue carefully prior to the study (S.A.S. and K.A.S).

### Microarray construction

The most representative areas of cancer cells and tumor stromal cells were identified. Using a tissue-arraying instrument (Beecher Instruments, Silver Springs, MD), two representative 0.6 mm core biopsies of cancer cells and two representative 0.6 mm core biopsies of tumor stromal cells were collected from each surgical specimen. As controls, normal lung tissue localized distant from the primary tumor was used in addition to samples from 20 normal lungs. All the cores were gathered in eight tissue microarray blocks (TMAs). Detailed methodology has been reported previously [15].

### Immunohistochemistry

**Figure 1:**
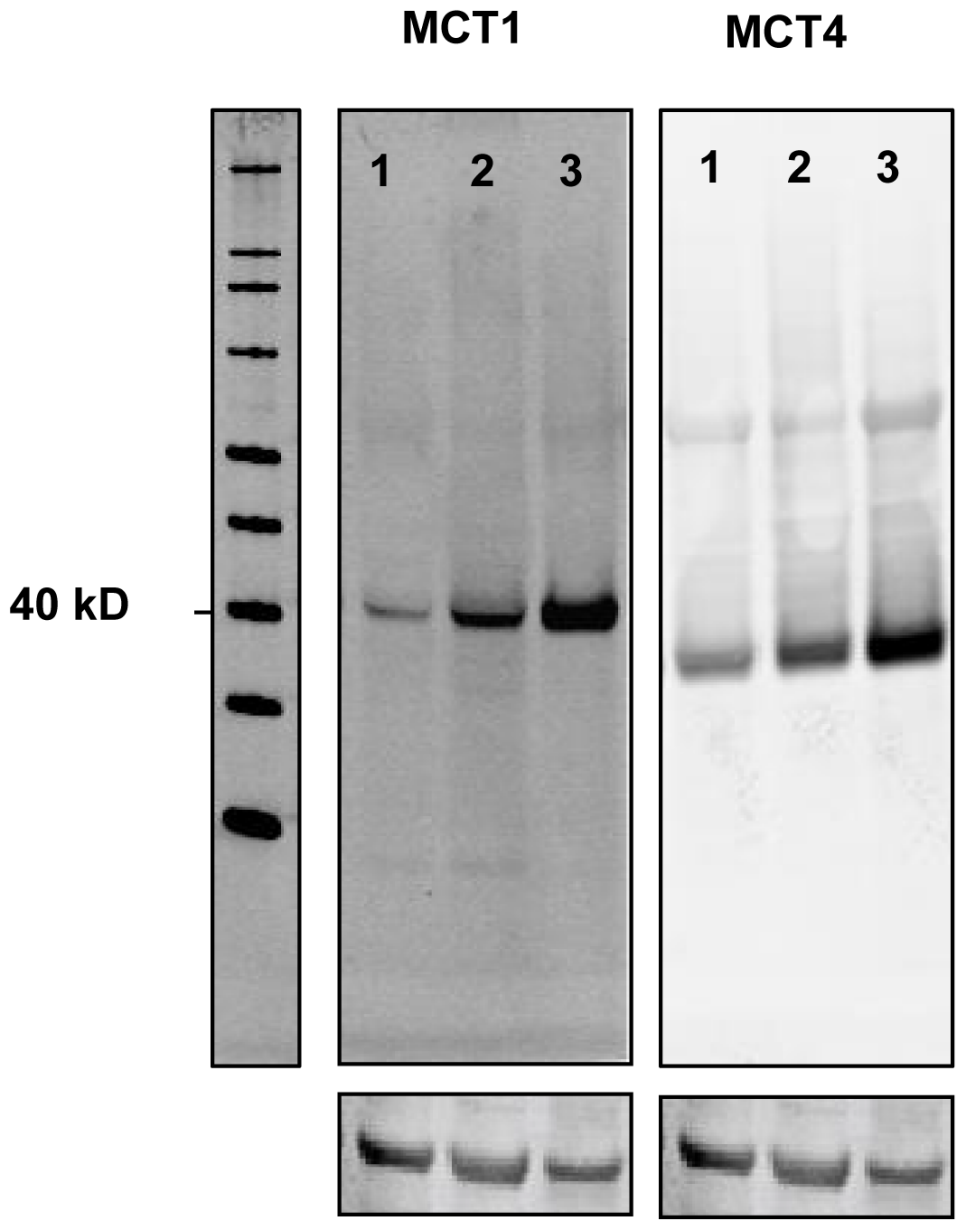
Western Blot of MCT1 and MCT4. In all cell lines investigated (A-549; lung adenocarcinoma, H661; large cell carcinoma, U-251 MG; neuronal glioblastoma) a protein band of approximately 40 kDa was detected corresponding to MCT1 and MCT4. Equal loading was ensured by B-actin.

All applied antibodies had been subjected to validation by the manufacturer for immunohistochemistry (IHC) on paraffin-embedded material, in addition MCT1 and MCT4 was validated by in-house Western blot analysis (Figure 1). All sections were deparaffinised with xylene and rehydrated with ethanol. The 4 µm sections containing tissue cores were subjected to the following antibodies: MCT1 (rabbit polyclonal, AB3538P, Millipore, 1/75), MCT2 (goat polyclonal, ab129290, Abcam, 1∶150), MCT3 (rabbit polyclonal, ab60333, Abcam, 1∶50), MCT4 (rabbit polyclonal, sc-50329, Santa Cruz, 1∶200) and GLUT1 (mouse monoclonal, AB40084, Abcam; 1∶500) [5].

MCT1 and MCT4 were stained using the Ventana Benchmark XT (Ventana Medical Systems Inc.) procedure ultraview DAB. Antigen retrieval was done automatic by CC1 mild (32 min).

For MCT2 and MCT3, antigen retrieval was done manually by placing the specimens in 0.01 M citrate buffer at pH 6.0 and exposed to microwave heating of 20 minutes at 450 W. The primary antibody was visualized by adding a secondary antibody conjugated with Biotin, followed by an Avidin/Biotin/Peroxydase complex (Vectastein ABC Elite kit from Vector Laboratories). Finally, all slides were counterstained with hematoxylin to visualize the nuclei.

### Scoring of immunohistochemistry

**Figure 2:**
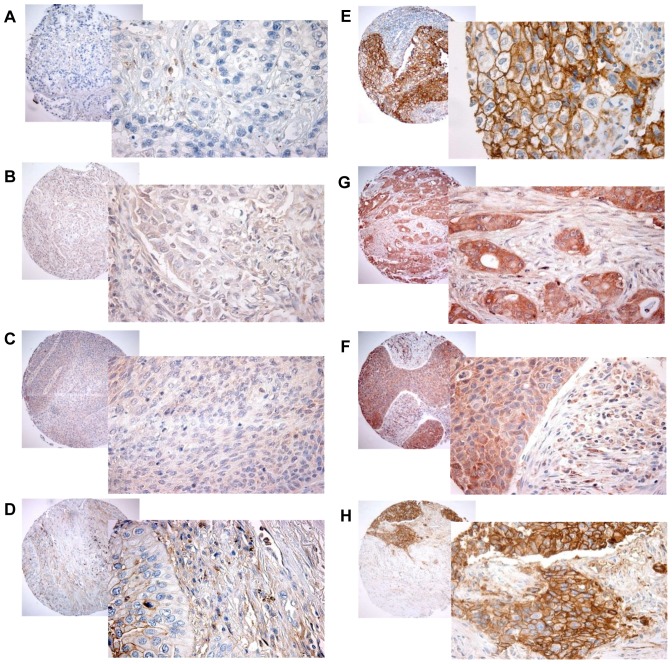
Immunhistochemical staining of MCT1–4 in NSCLC. Low expression: A) MCT1, B) MCT2, C) MCT3, D) MCT4. High expression: E) MCT1, F) MCT2, G) MCT3, H) MCT4. 100× and 400× magnification.

Scoring was done using light microscopy, and performed independently and semi-quantitatively by one experienced pathologist (S.A.S) and one M.D (M.E). Both intensity and density was scored when possible. The dominant staining intensity in cores of cancer cells and stromal cells was scored as; 0 = negative, 1 = weak, 2 = intermediate, 3 = strong (Figure 2). Staining density was scored as 0 = none, 1 = 1–10%, 2 = 11–50%, 3 = 51–100%. In case of disagreement, slides were re-examined and consensus was reached by the observers. Inter-individual variability in IHC-scoring in both cancer cells and stromal cells was evaluated on the current material.

Mean scores for cancer cell cores and stromal cell cores were calculated. In cancer cells, high expression was defined as: >1.5 for MCT1 and MCT3; >1 for MCT2; >2 for MCT4. Density was used for MCT1 and MCT4 in cancer cells, while MCT2 and MCT3 cancer cells intensity scores were used. In stromal cells, high expression was defined as: >1 for MCT1 and MCT3; >1.5 for MCT2 and MCT4. For MCT1 and MCT2 stroma intensity scores were used. For MCT3 and MCT4 stroma density scores were used. The same IHC scoring method has been utilized in previous studies from our group [15].

Furthermore, we constructed four co-expression variables. The first co-expression variable was created to test the potential synergistic impact when both GLUT1 (glucose import) [5] and MCT4 (lactate export) is expressed in cancer cells; GLUT1c+MCT4c. The other three co-expression variables assessed the hypothesized synergetic effect of metabolic co-operation between cancer cells and stromal cells; GLUT1c+MCT1 (lactate import)s, MCT4c+MCT1s and MCT1c+MCT4s. Kaplan Meier curves of the co-expression variables were made with the following stratifications low/low, other (low/high or high/low), high/high.

### Western blot

Cell lysates were incubated with NuPAGE LDS Sample Buffer (Life Technologies, USA) for 5 minutes at 85°C, sonicated briefly and run on a NuPAGE 4–12% Bis Tris Gel (Life Technologies, USA). Blotting was performed onto a Hybond nitrocellulose membrane (GE Healthcare) using the NuPAGE blotting system (Life Technologies, USA). The membrane was incubated with Odyssey blocking buffer (LI-COR Biosciences, Germany) for 1 hour at room temperature. Primary and secondary antibodies were diluted in the blocking buffer. Anti-MCT1 antibody (Millipore, USA, cat#AB3538p) was used in the dilution of 1∶500, anti-MCT4 in the dilution 1∶500 (Santa Cruz, USA, cat#SC-50329) and anti-actin (Sigma, cat#A2066) 1∶2000. IRDye CW secondary antibodies (LI-COR, Germany) were used in dilution 1∶10000. Molecular weight markers used were SeeBlue Plus 2 (Life Technologies, USA, cat#LC5925) and Magic Mark XP (Life Technologies, USA, cat#LC5602). Images were acquired on the ODYSSEY Sa Infrared Imaging System (LI-COR, Germany).

### Maintenance of cell lines

NCI-H661 cells (ATCC #HTB-183) were grown in RPMI-1640 media (21875-034, Gibco), A549 cells (ATCC # CCL-185) were grown in Ham's F-12K (Kaighn's) media (21127-022, Gibco), U-251 MG cells (Sigma-Aldrich #09063001) were grown in DMEM with 4,5 g/l glucose (41965-039, Gibco). All media were supplemented with penicillin, streptomycin and 10% fetal calf serum.

### Statistical methods

The SPSS 20.0 (Chicago, IL, USA) was used to perform the statistical analyses.

The Kaplan-Meier method was used for univariate analyses. The log-rank test was used to test the statistical significance between survival curves stratified by marker expression.

The endpoint of this study was disease-specific survival (DSS). DSS was calculated from the time of surgery to the time of lung cancer death. The cox regression analysis (backward stepwise) was used to test the independent impact of variables that were significant in the univariate analyses. In Model 1, MCT1–4 was tested simultaneously, while in Model 2 co-expression variables were tested one by one. The significance level for stepwise entry and removal was set at 0.05 and 0.10 respectively. P = 0.05 was considered statistically significant for all analyses.

## Results

### Patients characteristics

In Table 1, demographic, clinical and histopathologic variables are presented. The last DSS update was done in January 2011. The patients' median age was 67.1 years (range 28–85) and the majority of the cohort was male (76%). Ninety-six percent of the cohort was previous or present smokers. The median follow-up time of survivors was 99 months (range 9.8–189). The NSCLC tumors were divided in the following subgroups according to histology; 191 squamous cell carcinomas (SCC), 113 adenocarcinomas (AC) and 31 large-cell carcinomas (LCC).

**Table 1:** Prognostic clinicopathologic variables as predictors of disease-specific survival in 335 NSCLC patients (univariate analyses; log-rank test).

Characteristics	Patients N, (%)	Median survival (months)	5-year survival (%)	P
Age				.42
≤65 years	156 (47)	98	56	
>65 years	179 (53)	NR	60	
Sex				.22
Female	82 (24)	190	64	
Male	253 (76)	98	56	
Smoking status				.26
Never	15 (5)	19	43	
Previous	105 (31)	84	55	
Present	215 (64)	NR	60	
WHO Performance status				**.016**
0	197 (59)	NR	63	
1	120 (36)	64	52	
2	18 (5)	25	33	
Weight loss				.76
<10%	303 (90)	190	58	
>10%	32 (10)	98	57	
Histology				**.028**
Squamous cell carcinoma	191 (57)	NR	66	
Adenocarcinoma	113 (34)	54	46	
Large cell carcinoma	31 (9)	98	56	
Differentiation				**<.001**
Poor	138 (41)	47	47	
Moderate	144 (43)	190	65	
Well	53 (16)	NR	68	
Surgical procedure				**0.007**
Wedge + Lobectomy	243 (73)	190	62	
Pneumectomy	92 (27)	37	47	
p-Stage				**<.001**
pI	157 (47)	NR	72	
pII	136 (41)	62	51	
pIIIA	42 (12)	17	24	
T-status				**<.001**
1	85 (25)	190	75	
2	188 (56)	84	57	
3	62 (19)	25	37	
N-status				**<.001**
0	232 (69)	NR	67	
1	76 (23)	35	43	
2	27 (8)	18	18	
Surgical margins				.37
Free	307 (92)	190	59	
Not free	28 (8)	47	48	
Vascular infiltration				**.001**
No	284 (85)	190	62	
Yes	51 (15)	27	33	

NR, not reached.

### Expression of hypoxic markers and their correlations

MCT1 and MCT4 expression was mostly membranous, while MCT2 and MCT3 was mostly cytoplasmic (Figure 2). A moderate correlation was observed between density of cancer cell expression of MCT1 and intensity of GLUT1 expression (r = 0.38, P<0.001). Between clinicopathological factors and MCTs, a moderate correlation was observed only between density of MCT1 in cancer cells and histology (r = 0.484, P<0.001) with high expression in 58% of squamous cell carcinoma compared to 34% in adenocarcinoma.

### Univariate analysis

The significant prognostic clinicopathological variables were; WHO performance status (P = 0.016), histology (P = 0.028), differentiation (P<0.001), surgical procedure (P = 0.007), p-Stage (P<0.001), T-status (P<0.001), N-status (P<0.001) and vascular infiltration (P = 0.001) (Table 1).

**Figure 3:**
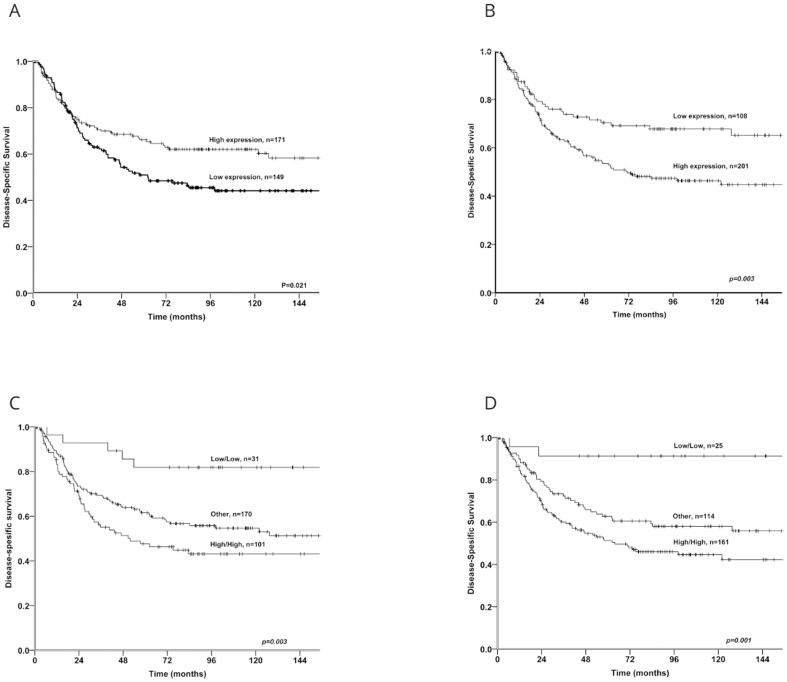
Kaplan Meier curves with DSS for expression of MCT1 and co-expression variables. Kaplan Meier curves with disease-specific survival for expression of monocarboxylate transporter (MCT)1 in cancer cells and stromal cells and the co-expression variable GLUT1c+MCT4c and GLUT1c+MCT1s in NSCLC. A) MCT1 in cancer cells, B) MCT1 in stromal cells, C) GLUT1c+MCT4c, D) GLUT1c+MCT1s.

Among the metabolic markers examined, ↑MCT1 expression in cancer cells (P = 0.021) and ↑MCT2 (P = 0.006) and ↑MCT3 (P = 0.020) expression in stromal cells correlated significantly with a favourable DSS (Table 2 and Figure 3). Whereas ↑MCT1 in stromal cells (P = 0.003) and ↑MCT4 in cancer cells (P = 0.027) were significantly associated with a poor DSS. MCT2 and MCT3 in cancer cells and MCT4 in stromal cells had no significant impact on survival.

**Table 2:** Monocarboxylate transporters (MCT) 1–4 in cancer and stromal cells as predictors of disease-specific survival in 335 NSCLC patients (univariate analyses; log-rank test).

Characteristics	Patients, N (%)	Median survival (months)	5-year survival (%)	P
**MCT1**				
Cancer cells				**.021**
High	171 (51)	NR	66	
Low	149 (44)	62	51	
Missing	15 (5)			
Stromal cells				**.003**
High	201 (60)	71	54	
Low	108 (32)	NR	70	
Missing	26 (8)			
**MCT2**				
Cancer cells				.364
High	220 (66)	127	58	
Low	67 (20)	NR	64	
Missing	48 (14)			
Stromal cells				**.006**
High	83 (25)	NR	72	
Low	231 (69)	127	55	
Missing	21(6)			
**MCT3**				
Cancer cells				.776
High	105 (31)	190	60	
Low	192 (58)	NR	59	
Missing	38 (11)			
Stromal cells				**.020**
High	277 (83)	61	190	
Low	32 (9)	40	27	
Missing	26 (8)			
**MCT4**				
Cancer cells				**.027**
High	132 (39)	NR	51	
Low	178 (53)	62	65	
Missing	25 (8)			
Stromal cells				.110
High	165 (49)	NR	62	
Low	139 (42)	71	53	
Missing	31 (9)			

The co-expression variables ↑GLUT1c+↑MCT1s (P = 0.001), ↑GLUT1c+↑MCT4c (P = 0.003) and ↑MCT4c+↑MCT1s (P = 0.009) were significantly associated with a poor DSS (Table 3). The co-expression marker ↑MCT1c+↑MCT4s (P = 0.006) was significantly associated with a positive DSS.

**Table 3:** Metabolic co-expression variables in cancer and stromal cells as predictors of disease-specific survival in 335 NSCLC patients (univariate analyses; log-rank test).

Co-expression variables	Patients N (%)	Median survival (months)	5-year survival (%)	P
**GLUT1c+MCT1s**				**.001**
Low/Low	25	NR	91	
Other	114	190	63	
High/High	161	64	51	
Missing	35			
**GLUT1c+MCT4c**				**.003**
Low/Low	31	NR	82	
Other	170	190	62	
High/High	101	52	48	
Missing	33			
**MCT4c+MCT1s**				**.009**
Low/Low	72	NR	74	
Other	129	98	58	
High/High	97	57	50	
Missing	37			
**MCT1c+MCT4s**				**.006**
Low/Low	81	62	51	
Other	114	62	51	
High/High	106	NR	72	
Missing	34			

### Multivariate analyses

Significant independent prognosticators for poor DSS in the NSCLC cohort were; T-status >1 (P = 0.002), N-status >0 (P = <0.001), moderate differentiation (P = 0.006), ↓MCT1 in cancer cells (HR: 1.9, CI 95%: 1.3–2.8, P = 0.001), ↓MCT2 in stromal cells (HR: 2.4, CI 95%: 1.5–3.9, P = <0.001) and ↓MCT3 (HR: 1.9, CI 95%: 1.1–3.5, P = 0.031), ↑MCT1 in stromal cells (HR: 1.7, CI 95%: 1.1–2.7, P = 0.016) and the co-expression variables ↑GLUT1c+↑MCT1s (HR: 7.3, P = 0.016) and ↑GLUT1c+↑MCT4c (HR: 3.3, P = 0.031) (Table 4).

**Table 4:** Results of Cox regression analyses (backward stepwise model) for clinicopathological factors and monocarboxylate transporters (MCTs) (model 1) and metabolic co-expression variables (*model 2).

Model 1	All patients N = 335		
Factor	HR	CI 95%	P
**T-status**			.002
T1	1(ref)		
T2	1.6	(0.95–2.7)	.079
T3	2.8	(1.6–5.1)	.001
**N-status**			.000
N0	1(ref)		
N1	2.0	(1.3–3.2)	.002
N2	2.8	(1.5–5.0)	.001
**Differentiation**			.006
Well	1(ref)		
Moderate	2.4		.007
Poor	1.4	(0.74–2.7)	.306
**WHO PS**	NS	NS	NS
**Vascular infiltration**	NS	NS	NS
**Histology**	NS	NS	NS
MCT1 Cancer cells^Total*^			.001
Low	1.9	(1.3–2.8)	
High	1(ref)		
**MCT1 Stromal cells**			.016
Low	1(ref)		
High	1.7	(1.1–2.7)	
**MCT2 Stromal cells**			.000
Low	2.4	(1.5–3.9)	
High	1(ref)		
**MCT3 Stromal cells**			.031
Low	1.9	(1.1–3.5)	
High	1(ref)		
**MCT4 Cancer cells**	NS	NS	NS
MCT4c+MCT1s*	NS	NS	NS
MCT1c+MCT4s*	NS	NS	NS
GLUT1c+MCT1s*			.016
Low/low	1(ref)		
Other	5.8	(1.4–24.4)	.016
High/high	7.3	(1.8–30.3)	.006
GLUT1c+MCT4c*			.031
Low/low	1(ref)		
Other	2.4	(.94–6.4)	.068
High/high	3.3	(1.2–3.3)	.016

We tested the PH-assumption (def.: the proportional hazards assumption; the relative hazard is constant over time, which is a requirement in the Cox proportional hazards model) for all markers, and for the MCT1-variable in cancer cells it was violated. Hence, the follow-up time was split into two intervals (>20 months, ≤20 months). We chose 20 months because the hazard was proportional past this point. We then performed a separate Cox regression analysis and the results were as follows: HR (total): 1.9, HR(>20 months): 2.3, HR(≤20 months): 0.9.

## Discussion

We present the first large-scale study on the prognostic role of MCT1–4 in both cancer cells and cells of the tumor stroma in NSCLC. Our main finding is that ↑MCT1 expression in cancer and stromal cells has a significant, independent impact on disease-specific survival, but with contrary effects in the two investigated compartments. ↑MCT1 in cancer cells is an independent positive prognostic factor. ↑MCT1 in stromal cells is an independent negative prognosticator. In addition, ↑GLUT1c+↑MCT1s and ↑GLUT1c+↑MCT4c show a substantial synergetic and independent impact on DSS when compared to low expression of these markers.

Our study confirms the presence of MCT1, MCT2 and MCT4 in NSCLC cancer cells and stromal cells, in agreement with the study by Koukourakis et al.[12]. To our knowledge, this is the first report on MCT3 being expressed in both cancer and stromal cells in NSCLC. We also show that MCT1 and MCT4 are located in the cell membrane, whereas MCT2 and MCT3 are expressed in the cytosol of NSCLC cells. The latter is in support of MCT2's hypothesized role in import of pyruvate in the mitochondria [11]. Besides, the specificity of the MCT1 and MCT4 antibodies was confirmed by Western blot, providing additional evidence for the validity of our main findings.

The association between ↑MCT1 expression in NSCLC cancer cells and improved survival was unexpected. Fang et al. reported in 2006 an elevated MCT1 mRNA expression to be correlated with a negative prognosis in neuroblastomas [16]. But apart from this study, a negative prognostic impact of MCT has only been demonstrated when MCT1 is co-expressed with CD147 or p53 [17]–[19]. Halestrap et al. reports that MCT1 is capable of transporting lactate both in and out of the cell, and that the direction of lactate transport is dependent on the pH-gradient [10]. And so, an explanation for our contrasting finding may be that MCT1 is transporting lactate in an opposite direction in neuroblastomas compared to NSCLC. MCT1 in NSCLC cancer cells may import lactate, while in neuroblastoma MCT1 exports lactate. In support of this, Chen et al. reported that lactate, likely imported by MCT1, can induce a certain gene expression profile in breast cancer, associated with a beneficial clinical outcome [20]. Some of these genes favored oxidative phosphorylation. For cells to be able to utilize lactate imported by MCT1, as a metabolic fuel, they must have oxygen available to enable oxidative phosphorylation and thereby ATP production. We hypothesize that ↑MCT1 expression in NSCLC cancer cells serve as a positive prognostic factor, because its expression indicates an overall less aggressive oxidative/metabolic cancer phenotype in NSCLC. However, functional studies are warranted to clarify MCT1's impact in NSCLC, since Izumi et al. stated that MCT1, together with MCT4, may promote cancer cell invasion in lung cancer [21].

Our data show that ↑MCT1 expression in stromal cells of the tumor is a negative prognostic factor in NSCLC, which is consistent with the finding of Sonveaux et al. [22]. They observed MCT1 expressed in endothelial cells to be involved in tumor angiogenesis activation. In their study, lactate activated the transcription factor HIF1α in endothelial cells, which promoted the expression of bFGF and VEGFR2. Vegran et al. state that lactate from cancer cells, exported by MCT4 and imported by MCT1 in endothelial cells, consecutively stimulate angiogenesis through NF-κB and IL-8 signalling [23]. In addition, Rattigan et al. found that lactate can induce MCT1 expression in mesenchymal cells, and in turn contribute to a metabolic co-operation of lactate homeostasis between recruited stromal cells and glycolytic cancer cells, which also is in agreement with our results [24].

Our data demonstrate that the ability to predict survival in NSCLC patients is substantially improved when we combine the key metabolic markers GLUT1 and MCT4, and GLUT1 and MCT1. Our study confirms that ↑GLUT1c+↑MCT4c has a negative prognostic impact in NSCLC, in agreement with the results of Meijer et al. [25]. However, they made their observation only in adenocarcinomas, while we found the same trend in all histological subgroups of NSCLC. This is most likely due to the fact that our NSCLC cohort is considerable larger than that of Meijer et al. To our knowledge, this is the first study reporting that co-expression of ↑GLUT1c+↑MCT1s has a significant synergetic, negative prognostic impact. This result is interesting, since it provides strong additional evidence of the theory of Koukourakis et al. [11], [12]. They hypothesized that stromal cells of the tumor is an accomplice in tumor growth and survival, by enabling cancer cells to maintain high glycolytic metabolism (↑GLUT1) by utilizing the by-product of glycolysis; lactate (↑MCT1 in stromal cells).

Cancer metabolism is regarded as a promising target for cancer therapy, and inhibition of MCT1 in cancer cells and in endothelial cells has been suggested as a potential target. So, is MCT1 a potential therapeutic target in NSCLC in light of our result? Despite being a positive prognostic marker when expressed in cancer cells, inhibition of MCT1 in NSCLC cancer cells will possibly not affect these less aggressive cells directly. Busk et al. report that inhibition of MCT1 leads to indirect starving of latent malignant hypoxic cancer cells that are present in the heterogenous tumor [26]. On the other hand, inhibition of MCT1 in cancer cells may be contraindicated since lactate import is thought to induce expression of a less aggressive gene expression profile [20]. Our data show that ↑MCT1 in stromal cells is a negative prognostic factor. Selective inhibition of MCT1 in stromal cells is a potential target strategy and inhibition of MCT1 in endothelial cells has already been suggested [22].

This is the first large-scale study on the prognostic role of MCT1–4 in NSCLC. The results presented herein demonstrate that MCT1 play crucial, but apparently opposing roles in cancer cell versus stromal cell compartments. We propose MCT1 as a new prognostic marker in NSCLC, although expression in cancer cells versus stromal cells mediates opposing prognostic impacts. Metabolic targeting is still largely an unexploited opportunity in cancer treatment more than 80 years after Warburg's ground-breaking studies. As MCTs are pivotal molecular effectors in tumor metabolism they serve as promising therapeutic targets. As there are contrasting prognostic impacts in cancer cells versus stromal cells, attention must be given to their role according to tumor compartments in future functional and expression analysis studies.
